# Genotyping analysis of the *Pax9* Gene in patients with maxillary canine impaction

**DOI:** 10.12688/f1000research.17147.1

**Published:** 2019-03-05

**Authors:** Evy Eida Vitria, Iwan Tofani, Lindawati Kusdhany, Endang Winiati Bachtiar

**Affiliations:** 1 Departement of Oral & Maxillofacial Surgery, Faculty of Dentistry,, Universitas Indonesia, Jakarta, 10430, Indonesia; 2 Departement of Prosthodontic, Faculty of Dentistry, Universitas Indonesia, Jakarta, 10430, Indonesia; 3 Department of Oral Biology, Oral Science Research Center, Faculty of Dentistry Universitas Indonesia, Jakarta, 10430, Indonesia

**Keywords:** Canine impaction, PAX9 gene, PCR, sequencing DNA, SNPs, Genotype

## Abstract

**Background**: Paired-box gene 9 ( *PAX9*) mutation is potentially associated with impaction in some patient populations. Here, we analyzed the relationship between *PAX* *9* polymorphism and the occurrence of maxillary canine impaction.

**Methods**: Patients with and without maxillary canine impaction were selected based on specific inclusion criteria, and samples of genomic DNA were obtained from a buccal mucosa swab. DNA was amplified by polymerase chain reaction and sequenced for further bioinformatics analysis to identify single nucleotide polymorphism (SNP) genotypes. Genotype and allele counting was performed in both case and control groups prior to conducting statistical analysis.

**Results**: Four SNPs were identified in patients with maxillary canine impaction, with relative confidence determined based on chromatogram-peak assessment. All SNPs were located in exon 3 of *PAX* *9* and in the region sequenced by the primer pair −197Fex3 and +28Rex3. Three of the SNPs (rs375436662, rs12881240, and rs4904210) were reported previously and are annotated in NCBI (dbSNP version 150), whereas another SNP mapped to chromosome 14 has not been reported. Patients with a CC genotype at SNP 3 [odds ratio (OR): 2.61 vs. TT; 1.28 vs. CT] and a CC genotype at SNP 4 [OR: 0.71 vs. GG; 0.79 vs. CG] were more likely to have maxillary canine impaction.

**Conclusions**: These results demonstrated that the presence of SNPs 3 and 4 is associated with increased likelihood of suffering from maxillary canine impaction.

## Introduction

Maxillary canine teeth are the second most common targets of impaction after the third molars ^1^. An impacted maxillary canine occurs in 1% to 3% of the general population and is twice as common in females as males ^2, 3^. It is commonly presented in clinics by patients often arriving with an aesthetic-related complaint.

Maxillary canine impaction in the palatal position is possibly caused by genetic factors and often accompanied by dental abnormalities in tooth shape, size, number, and structure. Abnormalities, such as agenesis, oligodontia, and peg-shaped teeth, have a genetic link to the presence of impacted teeth and generally manifest in developmental disorders during growth ^4– 8^. There is a relationship between malposition of certain teeth, such as palatal canines, and teeth agenesis. Similar to dental agenesis, canine-tooth-position anomalies affect several family members and are considered to be under strong genetic control ^9– 15^. Paired-box gene 9 ( *PAX9*) is most commonly involved in affecting the odontogenesis process and thought to determine the localization of tooth seeds ^16^. In this study, we identified an association between *PAX*9 genotype and the occurrence of maxillary canine impaction.

## Methods

### Patients

Patients were recruited from the Dental Hospital Faculty of Dentistry, Universitas Indonesia, and three different junior high schools in South Jakarta, and the study was conducted through clinical observations from May 2018 to August 2018. Those meeting the inclusion criteria (male or female, 10–25-years old, no systemic disease, and no hereditary disease) were either diagnosed with maxillary canine impaction (group I) or diagnosed as without (group II; control). Diagnosis was based on clinical examinations and radiographic interpretations performed by radiologists and orthodontists who were experts in their fields. Comprehensive clinical data were obtained for 132 patients (see Underlying data ^17^). All participants gave their written informed consent to participate in this study, which was approved by the Ethics Committee of the Faculty of Dentistry, Universitas Indonesia (No. 07107105/Ethical Approval/ FKGUI/2018).

### Polymerase chain reaction (PCR) amplification

Genomic DNA was collected from the buccal mucosa via swabbing and extracted using the Gene Jet whole blood genomic DNA purification kit (Cat. No. K0781; Thermo-Biogen, Karlsruhe, Germany). The area of the buccal mucosa/cheek to be treated was dried with a cotton roll to prevent salivary contamination. Samples from the buccal mucosa were obtained using a cytobrush (#C0104; Medscan; Cooper Surgical, Trumbull, CT, USA) on the bilateral buccal mucosa, with each side swabbed 10 to 15 times. The cytobrush swab was inserted into a screw-capped Eppendorf tube (Cat. No. SPL-60015; Extragene, Taichung City, Taiwan) containing 200 µL of 1% phosphate-buffered saline. Eppendorf tubes were labeled and stored in a freezer at −4°C. DNA concentration was measured using a Qubit Fluorometer 3 (#Q33216; Invitrogen, Carlsbad, CA, USA) at standard fluorescence wavelengths (excitation/emission: ~480/530 nm) with Qubits assay reagent (MP423-#Q32851; Qubit dsDNA HS assay kit; Invitrogen).

We used four sets of primers spanning the *PAX*9-coding region (exons 2, 3, and 4) ( Table 1) and standard PCR procedures for amplification of genomic DNA. PCR was performed using a T100 thermal cycler (No. #186-1096; Bio-Rad, Hercules, CA, USA) in a total volume of 25 µL containing 20 µL master mix (MyTag, 12.5 µL; Primer F, 0.5 µL; Primer R, 0.5 µL; and nuclease-free water, 6.5 µL) and 20 ng DNA. Samples were initially heated to 95°C for 10 min, followed by 30 cycles of denaturation at 95°C for 2 min, primer annealing at the optimal annealing temperature for the *PAX*9 primer for 1.5 min (optimal annealing temperature for *PAX*9 primers were as follows: F1-R1, 60°C; F2-R2, 64°C; F3-R3, 60°C; F4-R4, 56°C to 64°C), and extension/elongation at 72°C for 2 min then a final extension at 72°C for 15 min.

The PCR products were analyzed by electrophoresis using a 2% agarose gel (UltraPure Agarose; Cat. No. #16500500; Thermo Fisher Scientific, Waltham, MA, USA). To make the 2% agarose gel, 2 g agarose was added to 100 mL Tris base-acetate-EDTA (TAE) buffer, followed by the addition of 1 µL of GelRed nucleic acid gel stain (Cat. No. #41003-1; Biotium, Fremont, CA, USA). The wells were loaded with 5 µL of sample, with a DNA standard added to one well ; Cat. No. #SM0241; Thermo Fisher Scientific), and the gel was run at 100 V for 30 min.. The results of gel electrophoresis were visualized by using UV-transiluminator Gel DocTM 2000 (Cat. No. #170-8101; Bio-Rad, Hercules, CA,, USA).

**Table 1.** Primer sequences used in this study.

Gene	Primers	Sequence	Size (base pairs)
PAX9	-58F1ex2	AGGCAGCTGTCCCAAGCAGCG	410
	357R1ex2	GGAGGGCACATTGTACTTGTCGC	
	109F2ex2	ATCCGACCGTGTGACATCAGCC	525
	+10R2ex2	GAGCCCCTACCTTGGTCGGTG	
	-197Fex3	GGGAGTAAAACTTCACCAGGC	550
	+28Rex3	CCACCTGGCCTGACCCTC	
	-121Fex4	GGAGAGTAGAAGTCAGAGCATTGCTG	590
	+74Rex4	GAGACCTGGGAATTGGGGGA	

Extracted and adapted from Vastardis *et al*. (1996) and Nieminen *et al*. (2001). (+) indicates a sequence of DNA that is in front of the codon where transition begins and (−) the sequence of DNA that is behind the codonF, forward; R, reverse; ex, exon; numeration, nucleotide position in the sequence.

### DNA purification and sequencing

DNA from the PCR products was purified using an QIAquick PCR purification kit (Cat. No 28106; Qiagen, Hilden, Germany), and purified DNA was sequenced by First-Base Laboratories (Selangor, Malaysia). Sequencing data were edited using BioEdit software (v.7.0.9; Ibis Therapeutics, Carlsbad, CA, USA) and verified using NCBI BLAST (GenBank accession No. NG_0133557.1:5001-25240).

### Bioinformatics analysis

**Figure 1.**
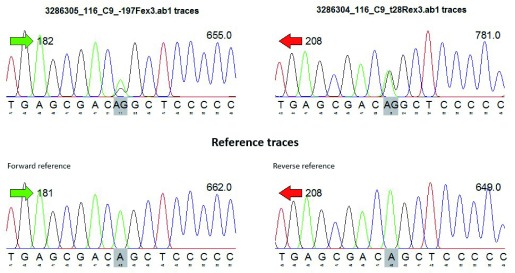
A representative chromatogram analysis of DNA sequencing assessment of data for sample 116 that reveals a heterozygous mutation corresponding to SNP rs3754366.

To detect sites containing potential single nucleotide polymorphisms (SNPs), sequencing data were converted to FASTQ format, and chromatogram-peak of each patient DNA sequence was performed as can be seen in the representative data from one sample ( Figure 1). Heterozygous SNPs were determined by visually identifying sites containing two overlapping peaks in both the forward and reverse sequences.

For each SNP identified, genotypes across all samples were obtained, annotated, and cross-checked against the NCBI SNP reference database ( dbSNP v.150). Both genotype and allele counting were performed before conducting statistical analysis for both the case and control groups.

### Statistical analysis

Statistical analysis of the data was performed using SPSS (v.20.0; SPSS, IBM Corp., Armonk, NY, USA). Analysis was initiated with a quality check of the variables. Analysis of genotype data was conducted using a chi-squared test to compare SNP frequency between patients with maxillary canine impaction and controls. A P < 0.05 was considered statistically significant.

## Results

### SNP identification

Four SNPs were identified, with all of these located in exon 3 of *PAX*9 sequenced using primer pair 3 (−197Fex3 and +28Rex3). Sequencing data for this region were available for 121 of 132 samples, and no SNPs were identified in regions sequenced using the other primer pairs.

Table 2 summarizes the number of SNPs identified. Of the four identified, three were reported previously in exon 3 of *PAX9* and are annotated in NCBI dbSNP (v.150; rs375436662, rs12881240, and rs4904210). The fourth SNP in exon 3 maps to chromosome 14, position 36,666,530, and has not been previously reported.

**Table 2.** SNPs identified according to the primers used.

Primer pair ID	Primer in Samples	Gene	Exon	No of SNPs Identified
Primer pair 1	110R2ex2	PAX9	2	0
	109F2ex2			
Primer pair 2	357R1ex2	PAX9	2	0
	-58F1ex2			
Primer pair 3	--197Fex3	PAX9	3	4
	+28Rex3			
Primer pair 4	-121Fex4	PAX9	4	0
	+74Rex4			

Table 3 lists the SNPs identified in this study along with their annotation/reference identification and the location of the nucleotide substitutions. Nucleotide changes included 640A>G in SNP 1, 700C>T in SNP 2, 717C>T and 718G>C in SNP 3.

**Table 3.** SNP annotations.

ID	Gene	Chromosome	Chromosome position	rsID	Substitution
SNP 1	PAX9	14	36,666,470	rs375436662	A>G
SNP 2	PAX9	14	36,666,530	?	C>T
SNP 3	PAX9	14	36,666,547	rs12881240	C>T
SNP 4	PAX9	14	36,666,548	rs4904210	G>C

### Genotype calling

Genotype assessment was performed for all of the identified SNPs, as well as controls, focusing on counts of homozygous (wild-type) and heterozygous and homozygous (mutant) alleles ( Table 4– Table 7).

**Table 4.** Genotype analysis of SNP 1 in case and control samples.

	Genotype Count			Allele Count	
SNP 1/rs375436662	AA	AG	GG	A	G
Maxillary Canine Impaction	82	1	0	165	1
Control	38	0	0	76	0
Total	120	1	0	241	1

**Table 5.** Genotype analysis of SNP 2 in case and control samples.

	Genotype Count			Allele Count	
SNP 2/rs?	CC	CT	TT	C	T
Maxillary Canine Impaction	82	1	0	165	1
Control	38	0	0	76	0
Total	120	1	0	241	1

**Table 6.** Genotype analysis of SNP 3 in case and control samples.

	Genotype Count			Allele Count	
SNP 3/rs12881240	CC	CT	TT	C	T
Maxillary Canine Impaction	46	32	5	124	42
Control	24	13	1	74	15
Total	80	45	6	198	57

**Table 7.** Genotype analysis of SNP 4 in case and control samples.

	Genotype Count			Allele Count	
SNP 4/rs4904210	CC	CG	GG	C	G
Maxillary Canine Impaction	24	42	17	90	76
Control	9	20	9	38	38
Total	33	62	26	128	114

For SNPs 3 and 4, all genotype and allele variations were observed in both the case and control samples, whereas for SNPs 1 and 2, the homozygous and heterozygous mutants were present in only one of the cases ( Table 6 and Table 7).

### Statistical analysis

We then analyzed associations between SNPs 3 and 4, which were present in both case and control samples, and maxillary canine impaction. We verified Hardy–Weinberg equilibrium (HWE) for both SNPs in case and control samples ( Table 8), with the TT genotype in SNP 3 (rs12881240) showing a higher degree of association with maxillary canine impaction than those with the CC genotype [odds ratio (OR): 2.61; 95% Confidence interval (CI): 0.29–23.61] or the CT genotype (OR: 1.28; 95% CI: 0.57–2.89), although this results was not statistically significant (P > 0.05). This result indicated a similar frequency of recessive and dominant carriers of SNP 3 between case and control samples. Furthermore, no statistical difference in allele frequency was observed between the two groups.

**Table 8.** Statistical analysis of associations between SNP and maxillary canine impaction.

Gene	SNP	HWE		Genotype Count		Genotype p-value	Allele Count		Allele p-value
	SNP 3/rs12881240	1	CC	CT	TT		C	T	
**PAX9**	Case		46	32	5	0.5831	124	42	0.3447
	Control		24	13	1		61	15	
	OR (95% CI)		1	1.28 (0.57-2,89)	2.61(0.29-23.61		1	1.38(0.71-2.68)	
	SNP 4/rs4904210	0.856	CC	CG	GG		C	G	
**PAX9**	Case		24	42	17	0.814	90	76	0.5427
	Control		9	20	9		38	38	
	OR (95% CI)		1	0.79(0.31-2.00)	0.71(0.23-2.16)		1	0.84(0.48-1.45)	

Chi-square test p<0,05 ( Sig 2- Tailed ), HWE: Hardy–Weinberg equilibrium

Individuals with the GG genotype in SNP 4 (rs4904210) were less likely to have maxillary canine impaction than those with the CC genotype (OR: 0.71; 95% CI: 0.23–2.16] and the CG genotype (OR: 0.79; 95% CI: 0.31–2.00] ( Table 8). Additionally, individuals were less likely to have the G allele than the C allele (OR: 0.84; 95% CI: 0.49–1.45]. However, similar to SNP 3, these results were not statistically significant (P > 0.05).

### Genotype efficacy for clinical diagnosis

The use of patient genotype information for clinical assessment represents a possible diagnostic strategy for predicting disease likelihood. Patient characteristics and genotype information were available from 121 of 132 samples, with Table 9 summarizing the clinical information associated with both the case and the control groups.

**Table 9.** Associations between gender and SNP frequency.

	SNP 3 / rs12881240				SNP 4 / rs4904210			
	CC (n=70)	CT (n=45)	TT (n=6)	P- Value	CC (n=33)	CG (n=62)	GG (n=26)	P- Value
**CASE** C Impaction	46	32	5	0.5831	24	42	17	0.814
Control	24	13	1		9	20	9	
OR (95% CI)	1	1.28 (0.57 -2.89)	2.61 (0.29-23.61)		1	0.79 (0.31-2.00)	0.71 (0.23 -2.16)	
**SEX** Male	28	21	4	0.3996	14	26	13	0.7725
Female	42	24	2		19	36	13	
OR (95% CI)	1	0.76 (0.36 - 1.62)	0.33 (0.06 - 1.94)		1	1.02 (0.43 - 2.40)	0.74 (0.26 - 2.07)	

Chi-square test p<0,05 ( Sig 2- Tailed )

Stratification of cases with SNP 3 (rs12881240) showed that 21 of 53 males and 24 of 68 females in the case group harbored the CT genotype, whereas only four males and two females harbored the TT genotype, with the difference between males and females in this group not statistically significant (P = 0.3996). Additionally, the OR according to gender was 0.76 (95% CI: 0.36–1.62) for individuals harboring the CT genotype of SNP 3 (rs12881240) relative to the CC genotype, whereas it was 0.33 (95% CI: 0.06–1.94) for individuals harboring the TT genotype relative to the CC genotype.

For SNP 4 (rs4904210), 26 of 53 males and 36 of 68 females in the case group had a CG genotype, whereas 13 males and 13 females had the GG genotype. Additionally, the OR according to gender was 1.02 (95% CI: 0.43–2.40) for individuals harboring the CG genotype of SNP 4 (rs4904210) relative to the CC genotype, whereas it was 0.74 (95% CI: 0.26–2.07) for individuals harboring the GG genotype relative to the CC genotype.

Although the frequency of genotype variation was higher in females than in males, the difference was not statistically significant (P = 0.7725). Consequently, these findings suggested that gender was not a major influence on the occurrence of maxillary canine impaction.

## Discussion

The etiology of canine impaction might be multifactorial and involve external factors, such as environmental input (e.g., trauma), local factors (e.g., lack of space, prolonged retention of primary teeth, trauma to permanent tooth seeds, rotation of permanent seed teeth, and the presence of pathological lesions, such as dentigerous cysts or odontoma), genetic factors, and systemic disease ^18– 24^. Dentistry is increasingly making use of genetic information, which plays an important role in addressing clinical problems, especially with regard to dental abnormalities or anomalies, including impaction of the maxillary canines. Previous studies show that tooth development is regulated by >200 genes ^23^. *PAX*9 encodes a transcription factor and is among the most frequently identified genes affecting the odontogenic process and involved in the occurrence of dental anomalies, such as agenesis teeth, congenital missing teeth, and variabilities in tooth size and position. The identification of genetic risk factors associated with canine impaction has recently become the subject of intensive research.

In this study, DNA sequencing of 121 of 132 patient and control samples identified four SNPs located in a similar region of *PAX*9 exon 3 ( Table 2). SNPs play a role in determining disease characteristics, including etiology and the incidence and risk of disease development. Subsequent analysis revealed that all of the identified SNPs would result in missense mutations ( Table 3). These findings suggest that SNPs might be efficacious for determining dental anomalies, specifically the impaction of maxillary canines.

Although we found no statistically significant association between *PAX*9 genotype and maxillary canine impaction ( Table 8), there were variations between patients with and without this condition according to the presence of SNP 3 and SNP 4, which carried a greater risk for maxillary canine impaction. A previous study reported that the genes involved in the impaction or displacement of canines into the palate are also responsible for controlling the growth and eruption of teeth ^25^, and Klein *et al*. ^26^ showed that dental anomalies (size, shape, and position of teeth and agenesis of teeth or supernumerary teeth) were determined by a set of genes involved in tooth development. The results of the present study suggested a potential role for *PAX*9 ** in tooth growth and development.

In summary, our findings showed no statistically significant association between SNP genotype and gender and demonstrated that although the frequency of impaction-related genotype variation in women was higher than that in men, the differences were not statistically significant. These results suggested that gender-associated variations in genetic profile do not contribute to the incidence of maxillary canine impaction.

## Consent

Written informed consent for publication of patient details was obtained.

## Data availability

### Underlying data

ABI files and chromatograms that support the findings of this study are available on reasonable request from the corresponding author [author initials] and by submitting the applicable request form (‘Form Padia’ to request access for genetic resources (available as part of the OSF deposit)). The data are not publicly available due to them containing information that could compromise research participant privacy.

Open Science Framework: Genotyping Analysis Pax9 Gene In Patients With Maxillary Canine Impaction. https://doi.org/10.17605/OSF.IO/B37CJ ^17^.

This project contains the following underlying data:

- ELECTROPHORESIS_ RESULTS- PAX9 GENE.docx_05-02-2018 (Gel images)
- RAW DATA _F1000 Vitria E 05-02-2019L.xlsx (Patient data)

### Extended data

Open Science Framework: Genotyping Analysis Pax9 Gene In Patients With Maxillary Canine Impaction. https://doi.org/10.17605/OSF.IO/B37CJ ^17^

This project contains the following extended data:

- PADIA form-Pax0 gene_Vitria E-05-02-2019.docx (Data access form)

Data are available under CC0 1.0 Universal Public Domain Dedication

## References

[1] Power SM Short MB: An investigation into the response of palatally displaced canines to the removal of deciduous canines and an assessment of factors contributing to favourable eruption. *Br J Orthod.* 1993;20(3):215–22. 10.1179/bjo.20.3.215 8399054

[2] Dachi SF Howell FV: A survey of 3,874 routine full-mouth radiographs. I. A study of retained roots and teeth. *Oral Surg Oral Med Oral Pathol.* 1961;14:1165–9. 10.1016/0030-4220(61)90204-3 13719264

[3] Thilander B Myrberg N: The prevalence of malocclusion in Swedish schoolchildren. *Scand J Dent Res.* 1973;81(1):12–20. 10.1111/j.1600-0722.1973.tb01489.x 4510864

[4] Wang Y Wu H Wu J: Identification and functional analysis of two novel *PAX*9 mutations. *Cells Tissues Organs.* 2009;189(1–4):80–7. 10.1159/000151448 18701815 PMC2824186

[5] Das P Stockton DW Bauer C: Haploinsufficiency of *PAX9* is associated with autosomal dominant hypodontia. *Hum Genet.* 2002;110(4):371–6. 10.1007/s00439-002-0699-1 11941488

[6] Kapadia H Frazier-Bowers S Ogawa T: Molecular characterization of a novel *PAX9* missense mutation causing posterior tooth agenesis. *Eur J Hum Genet.* 2006;14(4):403–9. 10.1038/sj.ejhg.5201574 16479262

[7] Pereira TV Salzano FM Mostowska A: Natural selection and molecular evolution in primate *PAX9* gene, a major determinant of tooth development. *Proc Natl Acad Sci U S A.* 2006;103 (15):5676–81. 10.1073/pnas.0509562103 16585527 PMC1458632

[8] Kapadia H Mues G D'Souza R: Genes affecting tooth morphogenesis. *Orthod Craniofac Res.* 2007;10(4):255–44. 10.1111/j.1601-6343.2007.00407.x 17973693

[9] Mercuri LG O’Neill R: Multiple impacted and supernumerary teeth in sisters. *Oral Surg Oral Med Oral Pathol.* 1980;50(3):293. 10.1016/0030-4220(80)90388-6 6965030

[10] Peck S Peck L Kataja M: The palatally displaced canine as a dental anomaly of genetic origin. *Angle Orthod.* 1994;64(4):249–56. 7978519 10.1043/0003-3219(1994)064<0249:WNID>2.0.CO;2

[11] Peck S Peck L Kataja M: Prevalence of tooth agenesis and peg-shaped maxillary lateral incisor associated with palatally displaced canine (PDC) anomaly. *Am J Orthod Dentofacial Orthop.* 1996;110(4):441–3. 10.1016/S0889-5406(96)70048-3 8876497

[12] Pirinen S Arte S Apajalahti S: Palatal displacement of canine is genetic and related to congenital absence of teeth. *J Dent Res.* 1996;75(10):1742–6. 10.1177/00220345960750100601 8955668

[13] Peck S Peck L: Palatal displacement of canine is genetic and related to congenital absence of teeth. *J Dent Res.* 1997;76(3):728–9. 10.1177/00220345970760030301 9109820

[14] Peck S Peck L Hirsh G: Mandibular lateral incisor-canine transposition in monozygotic twins. *ASDC J Dent Child.* 1997;64(6):409–13. 9466011

[15] Peck S Peck L Kataja M: Mandibular lateral incisor-canine transposition, concomitant dental anomalies, and genetic control. *Angle Orthod.* 1998;68(5):455–66. 9770104 10.1043/0003-3219(1998)068<0455:MLICTC>2.3.CO;2

[16] Neubüser A Koseki H Balling R: Characterization and developmental expression of *PAX9*, a paired-box-containing gene related to *PAX1*. *Dev Biol.* 1995;170(2):701–16. 10.1006/dbio.1995.1248 7649395

[17] Bachtiar EW: Genotyping Analysis Pax9 Gene In Patients With Maxillary Canine Impaction.2019 10.17605/OSF.IO/B37CJ PMC6489985 31069070

[18] Litsas G Acar A: A review of early displaced maxillary canines: Etiology, diagnosis and interceptive treatment. *Open Dent J.* 2011;5:39–47. 10.2174/1874210601105010039 21566691 PMC3091288

[19] Bishara SE: Impacted maxillary canines: A review. *Am J Orthod Dentofacial Orthop.* 1992;101(2):159–71. 10.1016/0889-5406(92)70008-X 1739070

[20] Thilander B Jakobsson SO: Local factors in impaction of maxillary canines. *Acta Odontol Scand.* 1968;26(2):145–68. 10.3109/00016356809004587 5247251

[21] Schindel RH Duffy SL: Maxillary transverse discrepancies and potentially impacted maxillary canines in mixed-dentition patients. *Angle Orthod.* 2007;77(3):430–5. 10.2319/0003-3219(2007)077[0430:MTDAPI]2.0.CO;2 17465649

[22] Richardson G Russell KA: A review of impacted permanent maxillary cuspids--diagnosis and prevention. *J Can Dent Assoc.* 2000;66(9):497–501. 11070629

[23] Becker A Sharibi S Chaushu S: Maxillary tooth size variation in dentitions with palatal canine displacement. *Eur J Orthod.* 2002;24(3):313–8. 10.1093/ejo/24.3.313 12143095

[24] Peck S Peck L Kataja M: The palatally displaced canine as a dental anomaly of genetic origin. *Angle Orthod.* 1994;64(4):249–56. 7978519 10.1043/0003-3219(1994)064<0249:WNID>2.0.CO;2

[25] NIH US National Library: What are single nucleotide polymorphisms (SNPs)? 2019 Reference Source

[26] Klein ML Nieminen P Lammi L: Novel mutation of the initiation codon of *PAX9* causes oligodontia. *J Dent Res.* 2005;84(1):43–7. 10.1177/154405910508400107 15615874

