# Supplementary material for: Genotyping analysis of the Pax9 Gene in patients with maxillary canine impaction
Source: F1000Res. 2019 Mar 5;8:254. [Version 1] doi: 10.12688/f1000research.17147.1 (PMC6489985; doi:10.12688/f1000research.17147.1)
Supplement: Peer review history

# Peer review: Reviewer response for version 1

**DOI:** 10.5256/f1000research.18746.r47551

**Published:** 2019-04-26

**Authors:** Fathilah Abdul Razak

**Affiliations:** 1 Department of Oral and Craniofacial Sciences, University of Malaya, Kuala Lumpur, Malaysia

The attempt to associate paired-box gene 9 ( *PAX9*) mutation to tooth impaction in patients was interesting and the decision to target on the occurrence of maxillary canine impaction was logical and appropriate. The study design was clearly outlined, well described and detailed. Data obtained was adequate, which enabled derivation of meaningful outcomes. The authors were able to demonstrate that the presence of SNPs 3 and 4 is actually associated with the increased likelihood of suffering from maxillary canine impaction. This finding definitely adds valuable information to the current knowledge.  

 In general, this is a nicely written manuscript. The flow of ideas and thoughts of the authors throughout the manuscript were effectively presented, focused and of high quality.

I have read this submission. I believe that I have an appropriate level of expertise to confirm that it is of an acceptable scientific standard.

# Peer review: Reviewer response for version 1

**DOI:** 10.5256/f1000research.18746.r45394

**Published:** 2019-03-22

**Authors:** Heni Susilowati

**Affiliations:** 1 Department of Oral Biology, Faculty of Dentistry, Gadjah Mada University, Yogyakarta, Indonesia

This study has reported new information that is very important to explain the effect of genes mutation in canine impaction. This research is very interesting. The researcher analysed the relationship between mutations and Pax9 gene polymorphisms with cases of canine tooth impaction. This research has been carried out using gene sequencing. The results showed an association between Pax9 gene mutations (SNPs 3 and 4) with the appearance of maxillary canine teeth impaction.

 The abstract contains concise and clear information about the background, objectives, methods, results and conclusions, so that it is quite easy for the readers.

 The following are inputs to improve the quality of this manuscript:

1. In the abstract, it is written in the background section: "Paired-box gene 9 (PAX9) mutation is often associated with impaction in some patient populations." This indicates that previous researchers may have conducted similar studies in different populations. It is advisable to write about this in the introduction, to strengthen the reasons for choosing the pax9 gene and also in the discussion, to analyse possible similarities or differences in pax9 polymorphism in different study populations.
2. The results showed that gender has no effect on the incidence of canine impaction, gender-associated genetic variation has no contribution. It would be better if the authors explain the possible causes, or show references from previous research reports that are relevant to the phenomenon. Does this occur only in canine impaction or is it possible for other tooth impaction cases?
3. Please add to the discussion section, factors that are likely to cause the emergence of Pax9 gene polymorphism.
4. Material and methods: the researcher conducted a sequencing analysis, please explain whether the method used is next generation sequencing.

I have read this submission. I believe that I have an appropriate level of expertise to confirm that it is of an acceptable scientific standard.
